# ELM: enhanced lowest common ancestor based method for detecting a pathogenic virus from a large sequence dataset

**DOI:** 10.1186/1471-2105-15-254

**Published:** 2014-07-28

**Authors:** Keisuke Ueno, Akihiro Ishii, Kimihito Ito

**Affiliations:** Division of Bioinformatics, Research Center for Zoonosis Control, Hokkaido University, Sapporo, Hokkaido, 001-0020 Japan; Hokudai Center for Zoonosis Control in Zambia, Research Center for Zoonosis Control, Hokkaido University, Sapporo, Hokkaido, 001-0020 Japan

**Keywords:** Next generation sequencing, Virus discovery, Diagnostic virology, Virome, Taxonomic identification

## Abstract

**Background:**

Emerging viral diseases, most of which are caused by the transmission of viruses from animals to humans, pose a threat to public health. Discovering pathogenic viruses through surveillance is the key to preparedness for this potential threat. Next generation sequencing (NGS) helps us to identify viruses without the design of a specific PCR primer. The major task in NGS data analysis is taxonomic identification for vast numbers of sequences. However, taxonomic identification via a BLAST search against all the known sequences is a computational bottleneck.

**Description:**

Here we propose an enhanced lowest-common-ancestor based method (ELM) to effectively identify viruses from massive sequence data. To reduce the computational cost, ELM uses a customized database composed only of viral sequences for the BLAST search. At the same time, ELM adopts a novel criterion to suppress the rise in false positive assignments caused by the small database. As a result, identification by ELM is more than 1,000 times faster than the conventional methods without loss of accuracy.

**Conclusions:**

We anticipate that ELM will contribute to direct diagnosis of viral infections. The web server and the customized viral database are freely available at http://bioinformatics.czc.hokudai.ac.jp/ELM/.

**Electronic supplementary material:**

The online version of this article (doi:10.1186/1471-2105-15-254) contains supplementary material, which is available to authorized users.

## Background

Most emerging infectious diseases are zoonoses, the pathogens of which are transmitted between humans and animals. The 2009 pandemic H1N1 influenza virus spread worldwide through reassortment that exchanged a gene segment between pigs and humans [1]. Recently, cases of influenza A virus H7N9 transmitted from birds to humans have been reported [2]. The 2003 severe acute respiratory syndrome (SARS) outbreak originated from the transmission of a novel bat coronavirus [3]. For the sporadically endemic Ebola virus, bats are suspected to be the natural reservoir, but this is still controversial [4]. Vector-borne zoonoses caused by transmission of viruses through mosquitoes and ticks have also become a public health concern. The 1999 outbreak of West Nile virus (WNV) that occurred in New York was caused by the transmission of the WNV among birds, horses and humans via mosquitoes [5]. Similarly, severe fever with thrombocytopenia syndrome (SFTS) was found to be due to a virus transmitted by ticks [6].

To prepare for the risk of emerging infectious diseases, we need to identify pathogenic viruses through surveillance of livestock and wild animals. Although universal PCR primers against 16S ribosomal RNA are available for the identification of bacteria, we needed specific PCR primers to identify viruses. In recent years, NGS technologies have become available for identifying novel viruses that cannot be found by Sanger sequencing due to the difficulty of isolation and passage culture [7].

The taxonomic classification of metagenomic sequences is an important task in NGS data analyses [8]. It has been widely applied to investigate the relationship between human health and the microbiome [9]. Recently, a metagenomic analysis of the virome in a monkey infected with simian immunodeficiency virus was conducted, suggesting that the virome was associated with enteropathy caused by HIV [10]. Through the first screening with NGS, the novel influenza virus H17N10 was identified in bats from metagenomic samples [11].

The taxonomic classification of NGS data uses sequence similarity searches such as BLASTX and BLASTN [12] to assign each sequence into a specific taxon based on the hits. However, with the similarity-based approach it is difficult to decide the resolution of assignments because the resolution depends on whether the sequences are conserved or species specific. The metagenome analyzer (MEGAN) employs the lowest common ancestor (LCA) concept in graph theory to estimate the taxonomical contents of samples [8]. MEGAN evaluates the resolution of similarity-based assignments as the level of taxonomy based on the LCA.

The LCA is the closest taxon shared among two or more taxa found by a BLAST search for a read. When multiple taxa are found by the BLAST search with sufficiently reliable BLAST scores, the common ancestor is a high-level taxon. The LCA assignments to high-level taxa are associated with conserved sequences. When a single taxon is found by a BLAST search for a read, the common ancestor still remains a low-level taxon. The LCA assignments to low-level taxa are associated with species-specific sequences. Thus, the LCA assignments to low-level taxa are more suitable for resolving closely related organisms than those to high-level taxa.

The SOrt-ITEMS [13] and CARMA3 [14] methods extended the LCA using a reciprocal BLAST search to reduce false positives in assignments. CARMA3 introduced the concept of the mutation rate into the LCA algorithm, and reinforced the reciprocal BLAST search to identify a novel taxon, relatives of which are numbered [14].

While taxonomic classification of metagenomic sequences has been developed with respect to accuracy, NGS technologies continue to improve sequencing throughput, and require considerable computational time and resources to perform taxonomic classification. The throughput of Roche 454 sequencing is 700 Mb with an average length of 400–800 bases. The present throughputs of NGS have become over 1 Gb with Illumina sequences of 600 Gb and an average length of ~100 bases, SOLiD sequences of 20 Gb with an average length of ~50 bases, and Ion Torrent PGM sequences of 1 Gb with an average length of ~200 bases [7]. These massive sequencing data prevent the fast detection of infecting viruses from metagenomic samples.

To reduce the computational time, we constructed a customized database composed only of viruses for the BLAST search. However, customized databases also increase accidental hits, i.e. the match of host sequences to viral genomic sequences. Here, we introduce ELM with a customized viral database for taxonomic identification. The method is based on the assumption that valid hits, the match of viral sequences to viral genomic sequences, raise the probability of finding other similar genomic sequences in the BLAST search. In other words, true assignments with the LCA should be sensitive to the threshold of the bit score in the BLAST search. Consequently, ELM can suppress the rise of false positive assignments while saving computational time and resources.

## Construction and content

**Figure 1:**
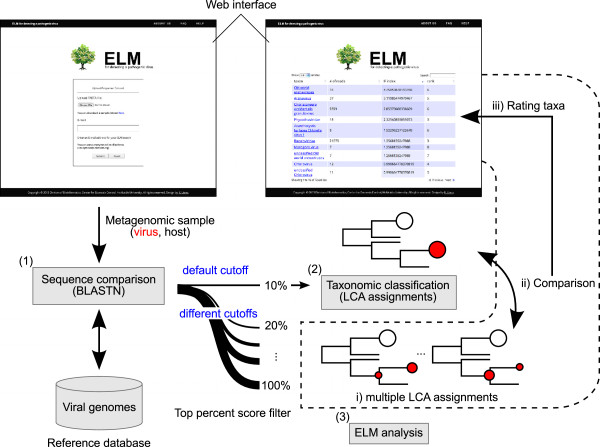
**Overview of ELM server and web interface.** Users input NGS data as a zip file (~1 Gb). (1) The web server matches NGS reads against known viral genomes using BLAST [12]. (2) Taxonomic classification based on the LCA is performed using MEGAN under the top 10% score filter [8]. (3) In ELM analyisis, multiple comparisons of the LCA assignments are performed under different top percent score filters. The server displays the results with the ratings of taxa.

The ELM server performs taxonomic identification of viral sequences from NGS datasets via three steps (Figure 1). In step one, the server carries out a BLASTN search for a customized database of viral genomic sequences. In step two, the server performs the LCA-based taxonomic assignments using MEGAN software [8] with default parameters. In step three, the server iterates the LCA assignments with different parameters for the threshold of the bit scores for the BLAST hits and investigates the taxa in which the number of assigned reads is significantly changed. In this step, the server provides a novel criterion for evaluating the LCA assignments.

### BLAST search for customized database

To reduce the computational time and save disk space, we constructed a customized database composed only of viral genomic sequences for a BLASTN search. First, the RefSeq genomic sequences were downloaded from the NCBI. Then a total of 3,336 viral genomic sequences were selected using a custom-made script program and converted into BLAST databases by the formatdb command in the NCBI BLAST package. We used the BLASTN program in the NCBI BLAST + version 2.2.26 package with the default parameters to search for similar sequences. The hits with an *E*-value under 10^-4^ were used for subsequent analyses.

### LCA analysis for taxonomic classification

The LCA method assigns sequence reads to taxa with a criterion for the resolution of assignments [8]. *h*(*q*, *s*) is the set of taxa found by a BLAST search for a sequence read *q* under the threshold of the bit score *s*. For a set of taxa *h*(*q*, *s*), the common ancestor located farthest from the root of the taxonomic tree defines the LCA as the representative taxon. Thus, the LCA allows the assignment of a read to a single taxon. At the same time, the taxonomic levels indicate the resolution of assignments because the LCA allows broad hits to be assigned as high-level taxa but specific hits to be assigned as low-level taxa. It also means that the number of the LCA assigned reads depends on the thresholds of the bit scores for BLAST hits.

We use MEGAN software version 4.62.5 for the LCA analysis [8]. MEGAN assigns sequence reads into taxa at different hierarchical levels such as family, genus, and species in the taxonomic ordering relation.

### ELM for evaluating the LCA assignments

**Figure 2:**
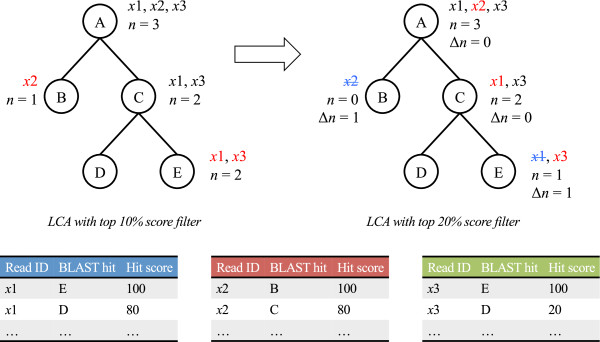
**Schematic representation of the ELM algorithm.** An example of the LCA assigned NGS reads into target viral taxa. The LCA assignment is affected by top percent score filters—that is, the BLAST hits for the similar sequences in the relatives. ELM evaluates this effect on the assignments. Circled A to E represent viral taxa on a taxonomic tree. The reads assigned as the LCA are shown in red. The reads corresponding to the reads assigned to descendant taxa as the LCA are shown in black. The total number of the LCA assigned reads for each taxon and its descendants is denoted as *n*. Δ*n* indicates the differences in *n* as varying thresholds of top percent score filters. The reads with strikeouts (blue) are the LCA assignments shifted into the upper taxon.

Since the LCA method solely provides the taxonomic levels of the assignments and the BLAST scores for short reads depend on local genomic regions, it is difficult to discriminate between true and false assignments with the LCA assignments or the BLAST scores alone. Here we combine the LCA assignments with top percent score filters for the BLAST hits to find out not only species-specific but also conserved reads, and introduce an additional criterion for the taxonomic assignment. First, ELM repeats the LCA analysis further under different top percent score filters for the BLAST hits and compares these LCA assignments with the reference assignment under the top 10% score filter (Figure 2). Here, the top *x* percent score filter retains the BLAST hits whose bit scores lie within *x*% of the best score [8]. *n*(*x*) is the total number of the LCA assigned reads for a taxon and its descendants under top *x* percent score filter. Then the difference Δ*n* from that under the reference top 10% score filter is given by:
1

Here, Δ*n* indicates to what extent the assigned reads are shifted into upper taxa as increasing *x* greater than 10%. We analyzed the increase of Δ*n*, which is associated with sequence similarity to relatives, to discriminate between true and false assignments. In the statistical analysis of Δ*n*, we introduce the inflation index *IF*, which is the *Z* score for outlier detection, to compare the effect of top percent score filters on the taxonomic assignments. The *IF* for a taxon is given by:
2

where *μ* is the average of Δ*n* for all assigned taxa, and *σ* is the standard deviation. Since multiple comparisons in *IF*s under top percent score filters ranging from 20% to 100% are performed nine times at 10% intervals, a *P* value of less than 0.05/9 is accepted for statistical significance after Bonferroni correction. Accordingly, *IF* >2.54 (one-tailed) is accepted with statistical significance.

## Utility and discussion

### Benchmark tests for NGS datasets

To evaluate the ability of ELM to detect pathogenic viruses from large sequence datasets, five real datasets were used. Dataset 1 consisted of 4,449,766 unassembled reads from a rodent sample in Zambia [15]. Reads with an average length of 236 bases were obtained by Ion Torrent Personal Genome Machine (PGM) sequencing. Dataset 2 consisted of 4,146,547 unassembled reads from a reptile sample (SRR: 527074) deposited in the NCBI Sequence Read Archive (SRA). Reads with an average length of 200 bases were obtained by Illumina sequencing [16]. Dataset 3 consisted of 12,393,506 unassembled reads from a simian sample (SRR: 167721) deposited in the SRA. Reads with an average length of 73 bases were obtained by Illumina sequencing [17]. We selected these three datasets to evaluate the effects of the read length, host and NGS platform. Furthermore, we applied ELM to fecal samples including multiple virus and phage taxa in dataset 4 (SRR: 1055974 for 12-day-old piglets) and dataset 5 (SRR: 1055972 for 54-day-old piglets). Reads with an average length of 291 bases in dataset 4 and 400 bases in dataset 5 were obtained by 454 GS FLX Titanium sequencing [18]. In these benchmark tests, the BLAST searches were performed on a workstation with an Intel Sandy Bridge CPU 2.6 GHz processor. We compared the result of the BLASTN search for the customized database with that for the NCBI NT database.

### Identification of infecting viruses using the LCA with BLASTN-NT

**Figure 3:**
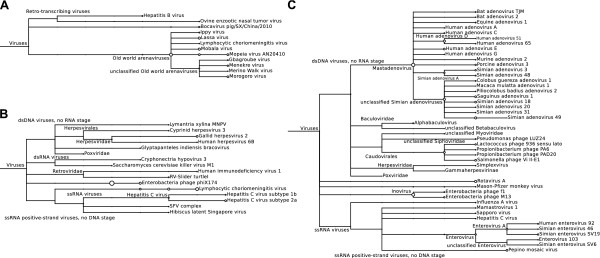
**Taxonomic identification using the LCA with BLASTN-NT.** The taxonomic trees for **(A)** rodent, **(B)** reptile and **(C)** simian samples. The circle sizes indicate the relative numbers of assigned reads. These trees were created using MEGAN [8]. Here, only the viral taxa are illustrated.

To identify infecting viruses, we performed conventional LCA-based assignment using the results of a BLASTN search of the NCBI NT database (Figure 3). The taxa assigned at the 6th taxonomic level from the root in dataset 1 showed that this rodent host was infected with *Old world arenaviruses* (Figure 3A). A previous study showed that the rodent host was infected with *Luna virus*, which belongs to the *Old world arenaviruses* [15]. Totally, 99.9% of the sequences were derived from eukaryotes, including sequences from the rodent host. The reptile host in dataset 2 was infected with *Lymphocytic choriomeningitis virus* (Figure 3B). This result was consistent with the closest virus described in the literature [16]. In dataset 2, 99.5% of the sequences were probably derived from the reptile host. According to the literature concerning dataset 3, the simian host was infected with a novel simian adenovirus, which is close to *Simian adenovirus 3*, *Simian adenovirus 18* and *Simian adenovirus 21* with about 55% pairwise nucleotide identity [17]. We found *Simian adenovirus 49*, *Simian adenovirus 18* and *Simian adenovirus 1* in dataset 3 (Figure 3C), suggesting results similar to those in the literature. Similarly, in dataset 3, most of the sequences (95.1%) were likely derived from the simian host.

To assess the required computational resources, we measured the elapsed time for the BLAST search (Table 1). As seen in Table 1, we found that the elapsed time for the BLAST search depended on the number of reads and hits. Although multiple threads and parallel jobs reduced the computational time, we needed at least one day with 8 threads and 32 parallel jobs. The sizes of the resulting tabulated format files ranged from 60–648 gigabytes, possibly affecting the elapsed time for the LCA analysis.

**Table 1:** **Elapsed time for the LCA with BLASTN-NT**

Dataset No.	# of reads	# of BLASTN hits	CPU time	
			BLAST	LCA
1	4,449,766	4,424,602	12,179 h	96 m
2	4,146,547	2,754,210	8,704 h	22 m
3	12,393,506	10,674,129	23,313 h	82 m

### Taxonomic classification using the LCA with BLASTN viruses

**Figure 4:**
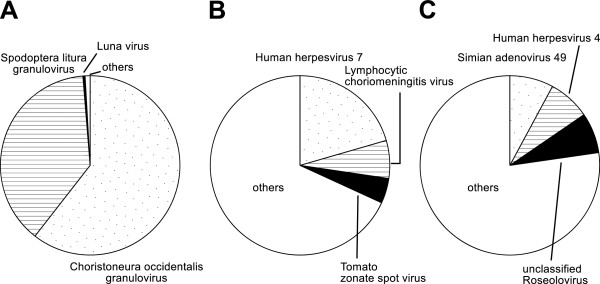
**Taxonomic classification using the LCA with BLASTN viruses.** The pie charts illustrate the number of reads assigned to taxa for **(A)** the rodent sample at the 6th taxonomic level, **(B)** the reptile sample at the 7th taxonomic level and **(C)** the simian sample at the 7th taxonomic level. Here, only the top three taxa are denoted.

According to the literature on taxonomic classification, the sequence similarity search of BLAST is a computational bottleneck [14]. Therefore, we used the customized viral database for a BLAST search to investigate how much the computational time was reduced and whether the conventional LCA could identify the infecting viruses. In Figure 4, the top 3 assigned reads show the capturing of infecting viruses. However, most of the assigned taxa were false positives (Figure 4). At the 6th taxonomic level of assignments in dataset 1, 98.7% of the reads were assigned into *Choristoneura occidentalis granulovirus* and *Spodoptera litura granulovirus*, and only 0.4% (1,518/383,939) of them were assigned into *Luna virus* (Figure 4A). In the case of BLAST-NT, we failed to identify *Luna virus* but detected *Old world arenaviruses*, with 1,245 reads at the 5th taxonomic level, including the following relatives: *Mobala virus*, 125 reads; *Morogoro virus*, 73 reads; and *Mopeia virus*, 56 reads. In dataset 2, 8,387 reads were assigned into 141 viral taxa at the 7th taxonomic level, and 573 were assigned into *Lymphocytic choriomeningitis virus* (Figure 4B). In the case of BLAST-NT, 454 reads were assigned into *Lymphocytic choriomeningitis virus*. These results showed consistency between BLAST viruses and BLAST-NT. Of the 5,952 reads assigned into viral taxa at the 7th taxonomic level in dataset 3, 468 were assigned into *Simian adenovirus 49* (Figure 4C). The most assigned taxon in BLAST viruses was *Simian adenovirus 49*, but 99 reads in BLAST-NT were assigned into the closest relative, *Simian adenovirus 18*. Although the assignments with BLAST-NT were more favorable than those with BLAST viruses, the coverage of the identified *Simian adenovirus 49* was sufficient to perform the subsequent analysis. These results showed that the sensitivity of the LCA with BLASTN viruses outperformed the LCA with BLASTN-NT, suggesting that the BLAST search of the viral database was sufficient for subsequent analysis.

Next, we investigated whether the elapsed time for the BLASTN search was effectively reduced (Table 2). The elapsed time in the BLAST search was reduced to 0.03%-0.05% (Tables 1 and 2). This showed the synergy effect of the reduction of the custom database to 0.1% (from 38 Gb to 49 Mb) for the size of FASTA files and to 1.4%-8.8% for the number of BLASTN hits (Tables 1 and 2). Furthermore, the elapsed time for the LCA analysis was also reduced despite the additional nine assignments for ELM analysis.

**Table 2:** **Elapsed time for ELM with BLASTN viruses**

Dataset No.	# of BLASTN hits	CPU time	
		BLAST	LCA
1	387,271	4 h	5 m
2	38,948	4 h	1 m
3	379,780	6 h	5 m

### Identification of infecting viruses using ELM with BLASTN viruses

**Figure 5:**
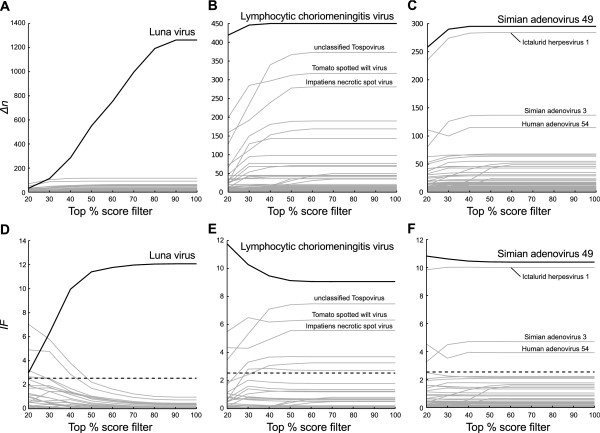
**ELM analyses of BLASTN viruses below the varietas level.** The solid lines depict the difference Δ*n* of the number of the LCA assigned reads for **(A)** rodent, **(B)** reptile and **(C)** simian samples from that under the reference top 10% score filter and the inflation index *IF* for **(D)** rodent, **(E)** reptile and **(F)** simian samples. *IF*s indicate how much Δ*n* deviates from a mean of that for all assigned taxa under each top percent score filter. The taxon described in the literature is shown as a bold line. *IF*s below the dashed lines indicate those under the null hypothesis.

To reduce the false assignments of the BLAST search for the customized viral database, we compared true and false assignments to confirm whether the true assignments altered into high-level taxa in ELM analysis (Figure 5). As shown for the 6th taxonomic level assignments of dataset 1 in Figure 5A and D, the assignment of *Luna virus* was significantly changed (*IF* >2.54, ranging from 20% to 100%), suggesting that ELM correctly identified the infecting virus. In the 7th taxonomic level assignments of dataset 2, the assignment of *Lymphocytic choriomeningitis virus* was most changed (*IF* >9, ranging from 20% to 100%) but, at the 6th taxonomic level, those of *unclassified Tospovirus*, *Tomato spotted wilt virus* and *Impatiens necrotic spot virus* were only slightly changed (Figure 5B and E). Figure 5C and F show that, in the 7th taxonomic level assignments of dataset 3, the assignment of *Simian adenovirus 49* was significantly changed (*IF* >10, ranging from 20% to 100%). However, at the 6th taxonomic level, the assignments of *Ictalurid herpesvirus 1*, *Simian adenovirus 3* and *Human adenovirus 54* were also changed, suggesting that ELM failed to exclude the false assignment of *Ictalurid herpesvirus 1*. On the other hand, ELM excluded the most of the false assignments (*IF* <2.54, ranging from 20% to 100%). These results suggested that we could find out true assignments mainly by high inflation indices and partly by low taxonomic levels. The results for viruses identified using ELM with BLASTN viruses were assembled using SSAKE v3.8.1 [19] and are summarized in Table 3.

**Table 3:** **Detection of the viral genomes using ELM with BLASTN viruses**

Dataset No.	Virus ^a^	# of reads	# of contigs	Average contig length
1	*Luna virus*	1,518	405	454 nt
2	*LCMV*	573	33	117 nt
3	*SAdV-49*	468	11	89 nt

Contigs were assembled using SSAKE v3.8.1 [19]. ^a^ *LCMV*, *Lymphocytic choriomeningitis virus*; *SAdV-49*, *Simian adenovirus* 49.

Next, we evaluated the effect of the BLAST hit score on the inflation indices. The results showed that the inflation indices had little association with the *E*-value in the BLAST search (Additional file 1: Figure S1). We also investigated the coverage of the BLAST hits. Valid hits in dataset 1 were distributed across target genomic sequences but not a specific genomic sequence, something not seen in datasets 2 and 3 (Additional file 1: Figure S2).

### Virome analyses using ELM with BLASTN viruses

To investigate whether ELM could detect multiple viral taxa, we analyzed the fecal virome of piglets using ELM with BLASTN viruses. We identified the shift of *Kobuvirus* in dataset 4 to *Bocavirus* and *Dependovirus* in dataset 5, which depended on the age of the piglets (Table 4). These results were consistent with abundant virus genera described in the literature [18]. However, we failed to identify *pig stool-associated small circular DNA virus* in dataset 5 (Table 4). This virus belongs to the *single-stranded circular DNA viruses*. The members of this family show extensive genetic diversity [20]. The results suggested that, in this case, the inflation index was not preferable for evaluating the LCA assignments.

**Table 4:** **Detection of abundant virus genera in fecal viromes of piglets using ELM with BLASTN viruses**

Dataset No.	ELM with BLASTN viruses (# of reads)	LCA with BLAST-NT (# of reads)
4	*Kobuvirus* ^a^ (6,449)	*Kobuvirus* (6,446)
5	*Dependovirus* ^a^ (759),*Bocavirus* (133)	*Dependovirus* (754), *Bocavirus* (528), *Chimpanzee stool associated circular ssDNA virus* ^b^ (106)

^a^The genus includes descendant taxa *IF* >2.54. ^b^According to the literature [18], this virus is the novel *pig stool-associated single-stranded DNA virus*, which is not assigned to a specific genus.

### Interpretation of results

ELM with a specific database drastically reduced the computational time and saved disk space. Furthermore, ELM was effective even for short reads. Though short reads can reduce the accuracy of BLAST searches, in this study we verified ELM for average lengths of between 73 and 400 bases. The results showed no difference between the capabilities for taxonomic assignment.

One approach to reduce the computational time needed for the BLAST search is the subtraction of reads by mapping host-derived reads onto reference sequences [21, 22]. This approach might be considered effective for reducing analyzed sequence data but is limited to known hosts. It is not suitable for surveillance of wild animals or metagenomic analysis because the host sequences have yet to be deposited in databases. Therefore, we need to decide a moderate threshold for NGS data before the mapping.

For ELM we adopted another approach using specific databases composed only of target sequences to reduce the computational cost. The difficulty in applying this approach directly to virus identification was the increase of false positive assignments (Figure 4). We tested several ways to solve this problem. As shown in Additional file 1: Figure S1, changing the threshold of the *E*-value dependent on the size of the database is probably not effective for discriminating between true and false assignments. The criteria for evaluating breadth coverage, i.e. the proportion of reads mapped across the hit genomes and the depth coverage (the number of reads mapped at a position), also failed to identify the target viruses (Additional file 1: Figure S2). On the other hand, ELM analyzed how sequence similarity to the relatives changes. This extension of the LCA method suppressed the rise in false positive assignments. A limitation of ELM would be the false-negative errors because ELM cannot detect viruses distantly related to other relatives (Table 4). Therefore, viruses without relatives should be carefully handled without the inflation index.

## Conclusions

ELM is especially useful for the first screening of infectious diseases caused by viruses. In surveillance for pathogenic viruses, taxonomic assignment of the host sequence is not necessary for the initial screening. For this, sensitivity for detecting viruses is particularly required. Our results suggest that ELM recovers most reads assigned to target viruses. Therefore, we can apply these results to further sophisticated analyses. ELM will contribute to analyses of NGS data for limited targets such as the direct diagnosis of viral infections.

## Availability and requirements

ELM is freely accessible at http://bioinformatics.czc.hokudai.ac.jp/ELM/. The web interface has been tested in the following web browsers: Google Chrome (version 36), Microsoft Internet Explorer (version 11) and Mozilla Firefox (version 31).

## Electronic supplementary material

Additional file 1: Figure S1: Distribution of *E*-values in BLASTN viruses. The figure shows the effect of *E*-value on discrimination between true and false assignments. **Figure S2.** Read coverage on the position of target genomes. The figure shows the effect of breadth and depth coverage on discrimination between true and false assignments. (PDF 2 MB)

## Abbreviations

NGS: Next generation sequencing
LCA: Lowest common ancestor
ELM: Enhanced LCA-based method.
