# Supplementary material for: ELM: enhanced lowest common ancestor based method for detecting a pathogenic virus from a large sequence dataset
Source: BMC Bioinformatics. 2014 Jul 28;15(1):254. doi: 10.1186/1471-2105-15-254 (PMC4124145; doi:10.1186/1471-2105-15-254)
Supplement: Supplementary file 1 — Additional file 1: Figure S1: Distribution of E-values in BLASTN viruses. The figure shows the effect of E-value on discrimination between true and false assignments. Figure S2. Read coverage on the position of target genomes. The figure shows the effect of breadth and depth coverage on discrimination between true and false assignments. (PDF 2 MB) [file 12859_2013_6522_MOESM1_ESM.pdf]

ELM: Enhanced lowest common ancestor based method for detecting a pathogenic virus from a large sequence dataset

Keisuke Ueno[1], Akihiro Ishii[2], Kimihito Ito[1]*

[1]Division of Bioinformatics, Research Center for Zoonosis Control, Hokkaido University, Sapporo, Hokkaido, 001-0020, Japan
[2]Hokudai Center for Zoonosis Control in Zambia, Research Center for Zoonosis Control, Hokkaido University, Sapporo, Hokkaido, 001-0020, Japan

*Corresponding author

Supplementary data

This file contains Supplementary Figures S1 and S2 with legends.

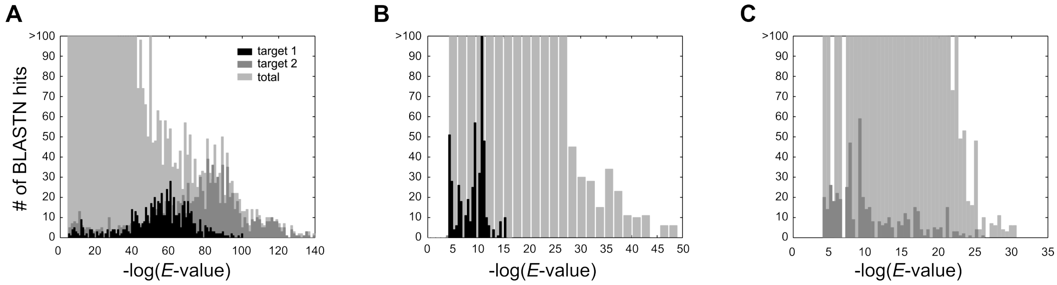

Figure S1. Distribution of *E*-values in BLASTN viruses for (A) rodent, (B) reptile and (C) simian samples.

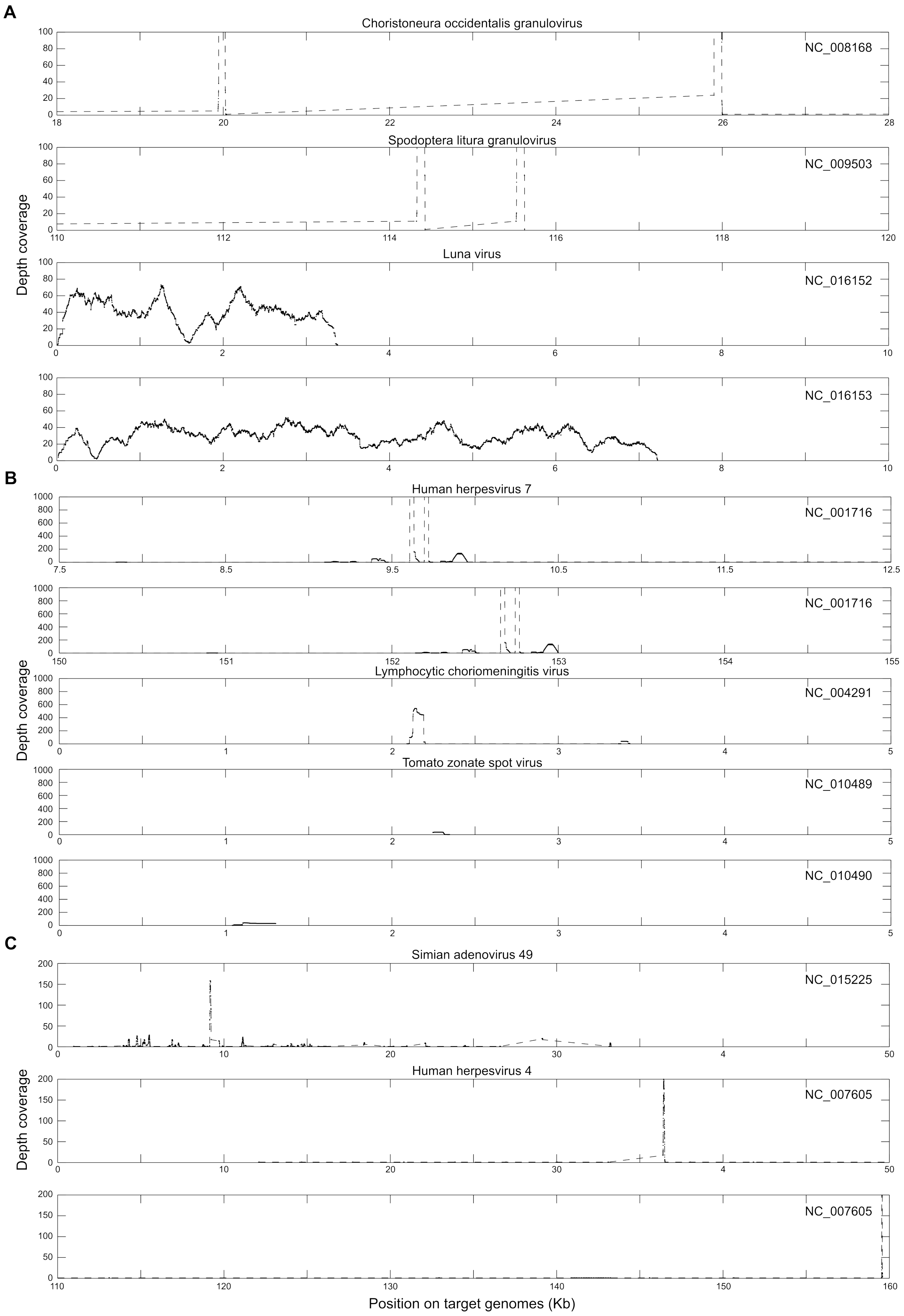

Figure S2. Read coverage on the position of target genomes for (A) rodent, (B) reptile and (C) simian samples.
